# High variability in SSU rDNA gene copy number among planktonic foraminifera revealed by single-cell qPCR

**DOI:** 10.1038/s43705-021-00067-3

**Published:** 2021-10-30

**Authors:** Tamara Milivojević, Shirin Nurshan Rahman, Débora Raposo, Michael Siccha, Michal Kucera, Raphaël Morard

**Affiliations:** 1 MARUM Center for Marine Environmental Sciences, University of Bremen, Leobener Strasse, 28359 Bremen, Germany; 2 Max Planck Institute for Marine Microbiology, Bremen, Germany

**Keywords:** Molecular biology, Molecular ecology

## Abstract

Metabarcoding has become the workhorse of community ecology. Sequencing a taxonomically informative DNA fragment from environmental samples gives fast access to community composition across taxonomic groups, but it relies on the assumption that the number of sequences for each taxon correlates with its abundance in the sampled community. However, gene copy number varies among and within taxa, and the extent of this variability must therefore be considered when interpreting community composition data derived from environmental sequencing. Here we measured with single-cell qPCR the SSU rDNA gene copy number of 139 specimens of five species of planktonic foraminifera. We found that the average gene copy number varied between of ~4000 to ~50,000 gene copies between species, and individuals of the same species can carry between ~300 to more than 350,000 gene copies. This variability cannot be explained by differences in cell size and considering all plausible sources of bias, we conclude that this variability likely reflects dynamic genomic processes acting during the life cycle. We used the observed variability to model its impact on metabarcoding and found that the application of a correcting factor at species level may correct the derived relative abundances, provided sufficiently large populations have been sampled.

## Introduction

The goal of community ecology is to understand the role of biotic and abiotic factors that control the variation of community composition in time and space [1]. This requires sampling over spatial, temporal and environmental gradients, across which the constituent taxa are identified and their abundance enumerated. This poses numerous challenges, such as the difficulty to cover the full range of organism sizes, in the open ocean spanning twelve orders of magnitude [2], with a single sampling protocol [3], or the difficulty to taxonomically identify each collected specimen in the plankton that spans the entire tree of life [4]. A potentially powerful workaround to these limitations is to analyse DNA in bulk environmental samples. By sequencing short informative DNA barcodes one can potentially produce an exhaustive inventory of all organisms in a given sample. No time-consuming sorting of single specimens and expert taxonomic assessment is involved in the process, providing fast access to large amounts of data. Indeed, this approach has been successfully applied to assess diversity in the sunlit ocean [5], abyssal plains [6], neotropical soils [7], for monitoring of pollution impacts [8] and a broad range of other settings and applications [9].

Metabarcoding, however, is not free of limitations. It needs robust experimental design [10] and only provides information on relative proportion (compositionality) of sequences, without knowing how they relate to the actual proportion of individuals in the community [11]. The choice of the amplified fragment is always a compromise between the target group (taxonomic coverage) and the specificity (taxonomic resolution), and the identification of species relies on the availability of curated reference databases that link the sequences to known taxa [12]. The small subunit ribosomal DNA (SSU rDNA) is a widely used gene for metabarcoding, because it is present in all eukaryotes and well documented in reference databases [13]. The SSU rDNA is a multi-copy gene, whose gene copy number has been studied in many other protist groups, such as dinoflagellates, chlorophytes, ciliates and stramenopiles [14–17]. There is a broad agreement that the gene copy number correlates with body size when considering organisms spanning several orders of magnitude in body length (See Figure W3 in [5]). The relationship to body size is clearly visible across the full range of cell and body sizes of eukaryotes, but it is less obvious at the scale of size variability within a single taxa or clade. For example, ciliates harbour much higher gene copy number than other protists compared to their size [15, 18] and small sized ciliates taxa can have higher gene copy number than large sized ones [19], contradicting the general rule of correlation between size and gene copy number. This is important, because differences in gene copy number that cannot be related to body size may bias community composition data obtained from metabarcoding studies in unpredictable directions [20].

In this study, we assess the number of gene copies of planktonic foraminifera, a group of mixotrophic protists with calcite shells that are widely used as paleoceanographic proxies [21]. They have pervasive cryptic diversity [22–24] but their biological diversity is well constrained and limited, as confirmed by metabarcoding studies [25, 26]. The molecular diversity of benthic foraminifera has been extensively documented with metabarcoding [6, 27–31] and previous work showed that the number of SSU rDNA gene copies varied between ~10 000 and ~30 000 between species [32]. However, the relationship to cell size has not been constrained because the analyses were based on pooled samples. Establishing a calibration between the number of gene copies and the number of individuals will increase the amount of information that could be extracted from metabarcoding data. Specifically, it will allow us to translate proportions of taxa represented by reads in metabarcodes to the proportions of individuals as recorded by census counts. In this way metabarcoding observations can be linked to the wealth of existing data on species occurrence and abundance, collectively allowing us to constrain the horizontal and vertical distribution of species and their relation to abiotic factors. This knowledge is necessary to interpret observations of changes in community composition in fossil assemblages [33]. To this end, we applied single-cell real time qPCR to characterize the variability in SSU rDNA gene copy number in five species of planktonic foraminifera and evaluate the relationship of gene copy number with individual size.

## Material and methods

### Sample collection

All specimens of this study were sourced from our collection of cryopreserved specimens available at the University of Bremen. Our collection contains specimens recovered from multi-net samples collected between 0 and 700 metres using nets with mesh sizes from 63 to 200 μm. The specimens isolated from the plankton were cleaned with brushes and transferred onto cardboard slides and air-dried before being stored at −20 °C or −80 °C until further processing. Further details of the collection procedures are described in [34]. We selected specimens of the morphological species *Globigerinella siphonifera*, *Trilobatus sacculifer*, *Neogloboquadrina pachyderma, Neogloboquadrina dutertrei* and *Globigerinita glutinata* collected during the cruises MSM66, M113/2, M124 and M133 at six stations. Specimens were identified under a Zeiss V8 stereomicroscope and those selected were photographed using a KEYENCE VHX 6000 digital microscope in standard position to produce focus stacked 2.5D images to quantify individual cell volumes (see below). After imaging, each specimen was transferred into individual 1.5 ml tube with 50 µl of DOC lysis buffer for single cell DNA extractions. Every 24th tube was left empty to serve as negative control.

### DNA extraction

The DOC lysis buffer [34] was chosen for this study because it dissolves entirely the shell of foraminifera and does not require purification steps that would lead to the loss of genetic material. The tubes were first sonicated in an ultrasonic bath for three minutes to break the shell and facilitate the digestion of the entire cellular material. Then, the samples were vortexed and shortly centrifuged to homogenize the buffer before being heated and vortexed at 60 °C for 1 h in an Eppendorf Thermomixer. Every 20 min the tubes were taken out of the thermomixer, vortexed and shortly centrifuged to ensure the homogeneity of the buffer throughout the digestion process. In case there were still cellular material visible after the end of digestion, the tubes were sonicated for two minutes again and the extraction was repeated until all material was dissolved. After the extractions were finished, the samples were stored at 4 °C until further processing.

### Volumetric data

Volumetric data of each specimen were extracted from the 2.5D pictures using a custom MATLAB script. The focus-stacked images taken with magnifications ranging between x200 and x500 yielded elevation maps with effective voxel side lengths between 2.05 and 0.82 µm. Foraminifera outline detection was performed by active contour segmentation and where necessary additionally manually edited when attachments, discolorations or shadows hampered automatic segmentation. Test volume was approximated by complementing the measured volume of the visible upper part of the specimen above mid outline height by an artificial spherical lower part. This artificial volume had the shape of the foraminifera outline at average outline height and decreased its outline parabolically until converging with height zero at the location of the centroid of the foraminifera outline height cross section (See Fig. S1).

### Primer selection

For accurate quantification of gene copy numbers it is recommended to use fragments of a length not exceeding 300 bp, optimally 50–150 bp long [35]. For foraminifera, the 37 F (38–132 bp) fragment qualifies but unfortunately not all planktonic foraminifera species can be successfully amplified using a single pair of primers. This is due to a 1–3 nucleotide difference on the position of the forward S14F1 primer between the spinose clade and other foraminifera mutations [36]. Alternatively, the pair of primer S18F-S19F that amplifies the fragment 45E–47F (179–312 bp) works for all planktonic foraminifera species but its length makes it a less ideal choice for qPCR amplification.

To assess the impact of both potential issues, we amplified the 37 F region by use of the primer pair S14F1-S15r2 for the species *Neogloboquadrina pachyderma* and the primer pair S14p-S15r2 for the species *Trilobatus sacculifer*. We also amplified the region 45E–47F using the primer pair S18F-S19F for both species. We compared the results by doing a linear correlation to assess the scalability of the results with both markers and proceeded with the pair S18F-S19F for quantification in all species (see results).

### PCR test

DNA extraction success was assessed with normal PCR using the S18F-S19F primer pair. Preparation of PCR master mix was handled under a sterilized UV hood. Master mix was composed as follows: 8.35 µl of Milli-Q water, 3 µl of 5x Phusion Green HF buffer (x1.00 final concentration), 0.75 µl of 50 mM MgCl2 (2.5 µmol/µl), 0.45 µl of 100% DMSO (0.75 µmol/ml), 0.30 µl of dNTP mix (0.20 µmol/ml), 0.5 µl of forward primer (0.33 µmol/ml), 0.5 µl of reverse primer (0.33 µmol/ml) and 0.15 µl of Phusion Green Hot Start II HF 2 U/µl polymerase (0.02 U/µl) and 1 µl of DNA extraction. The DNA was amplified with an initial denaturation at 98 °C for 30 s, followed by 35 cycles at 98 °C for 10 s, 65 °C for 30 s and 72 °C for 30 s and 10 min of final extension at 72 °C. Success in amplification was checked with agarose gel electrophoresis and only samples displaying strong single bands were retained for further analyses.

### Standards curve for qPCR

Prior to the quantification of gene copy numbers, standard curves were developed for each species. Positive PCR products obtained during the previous step were purified using the QIAquick PCR purification kit (QIAGEN) and the DNA concentration of the products were measured using the Quantus Fluorometer with Promega QuantiFluor dsDNA System kit following manufacturer’s instructions. The number of gene copies were calculated from the formula of [17]:

$${{{{{{{\mathrm{molecules/\mu l}}}}}}}} =	 \,{{{{{{{\mathrm{DNA}}}}}}}}\;{{{{{{{\mathrm{concentration}}}}}}}}\left( {{{{{{{{\mathrm{g/\mu l}}}}}}}}} \right)/\left( {{{{{{{{\mathrm{fragment}}}}}}}}\;{{{{{{{\mathrm{length}}}}}}}}\;{{{{{{{\mathrm{x}}}}}}}}\;660} \right)\\ 	 {{{{{{{\mathrm{x}}}}}}}}\;6.022 \times 10^{23}$$

We used the PFR² database [37] to establish the size of the fragments 45E–47F for every species and the fragment 37 F for the *N. pachyderma* and *T. sacculifer* (It includes the hypervariable region and the conserved flanking region). For the region 45E–47F the fragment sizes were the following: 385 bp for *Neogloboquadrina pachyderma*, 362 bp for *Trilobatus sacculifer*, 334 bp for *Globigerinella siphonifera*, 373 bp for *Neogloboquadrina dutertrei* and 413 to 420 bp for *Globigerinita glutinata*. For the 37 F region the fragment sizes were 212 bp for *N. pachyderma* and 177 bp for *T. sacculifer*. Series of 10-fold dilution from 10^−1^ to 10^−8^ were prepared and the number of gene copies in each dilution were calculated based on the numbers inferred from the initial PCR product divided by the dilution factor. We retained the dilution from 10^−3^ to 10^−8^ for each series because the less diluted samples would have been out of the range of the measured samples.

The quality of each dilution series was estimated using an optimized qPCR protocol. The protocol was optimized to use consumables conservatively while retaining maximum reproducibility of the results. We used the PowerTrack SYBR Green Master Mix (ThermoFisher Scientific) and used the following mix: 0.25 µl of Yellow Sample Buffer, 5 µl of PowerTrack SYBR Green Master Mix, 0.25 µl of forward and reverse primer (10 ng.µl^−1^), 3.25 µl of nuclease-free Milli-Q water and 1 µl of DNA template to a final volume of 10 µl. The qPCR was performed with the QuantStudio 1 Real-Time PCR thermocycler (Applied Biosystems, Thermo Fisher Scientific) with the following steps: initial denaturation at 95 °C for 2 min followed by 40 cycles of denaturation at 95 °C for 15 s and annealing-extension at 60 °C for 1 min and final Melt Curve Stage where the temperature ramped up from 60 °C to 95 °C increasing by 1 °C every 0.15 s. We retained as standard curves those having a R² equal or above 0.99, a single peak in the melt curve plot and an efficiency between 90% and 110 %.

### qPCR and final validation

After validation of the standard curves, all positive extractions were quantified with the following approach. Each qPCR session included only standards and samples belonging to the same species and marker, where samples and standards were loaded in triplicates to quantify experimental error, and every session included negative controls of extractions and negative PCR controls. The same optimized protocol presented above was used for every species and marker. After the end of the reaction, one triplicate of each sample and the negative controls were migrated to confirm that only a single fragment of the expected length was amplified in each reaction. As a final confirmation that the right target and species has been successfully amplified, another triplicate was purified with QIAquick PCR purification kit (QIAGEN) and sequenced by an external provider (LGC Genomics, Berlin). The obtained sequences were compared to the PFR² database and in case the amplified product was not belonging to the expected target, the data was discarded. Because of their short length and limited use outside of the purpose of this study, the sequences are provided as part of Supplementary Information but not deposited on NCBI.

### Data analyses

Prior to downstream analyses, the data were evaluated to exclude potentially inaccurate quantifications. We excluded quantifications where one replicate showed a significant deviation from the two other (automatically detected by the QuantStudio 1). Then, the gene copy numbers quantified in every reaction were multiplied by 50 to calculate the number of gene copies in every foraminifera cell (As 1 µl of DNA extract was used from the 50 µl of the entire extraction where each cell was dissolved). Next, the minimal number of gene copies per reaction was set to 100 copies, which would mean that there were at least 2 copies per 1 µl of DNA template, to prevent usage of single cell amplification results that are too close to the lower detection limit of the thermocycler. Quantification data are provided in Supplementary Information.

**Fig. 1:**
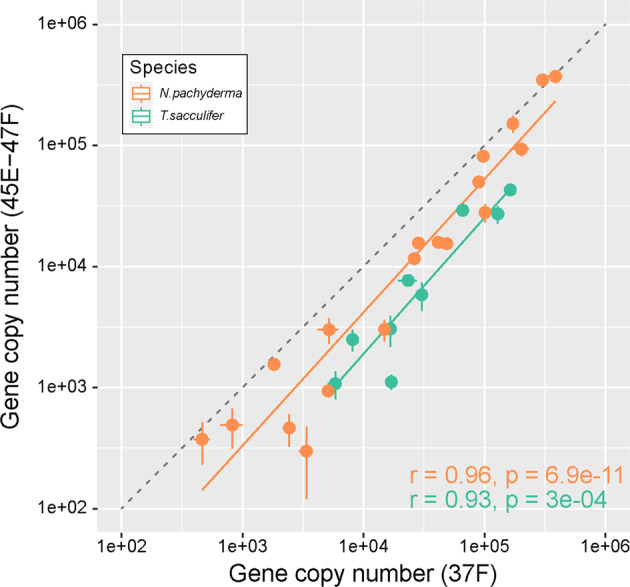
Linear regression between quantification of gene copy number made with the marker 37 F and 45E-47F on same specimens of the species *Neogloboquadrina pachyderma* and *Trilobatus sacculifer*. Each dot represents the average of triplicated quantification and the vertical and horizontal lines represent the standard deviation of the measurements. The dashed line indicates the expected 1:1 relationship and the inferred relationships are offset towards higher quantification by the 37 F region.

**Fig. 2:**
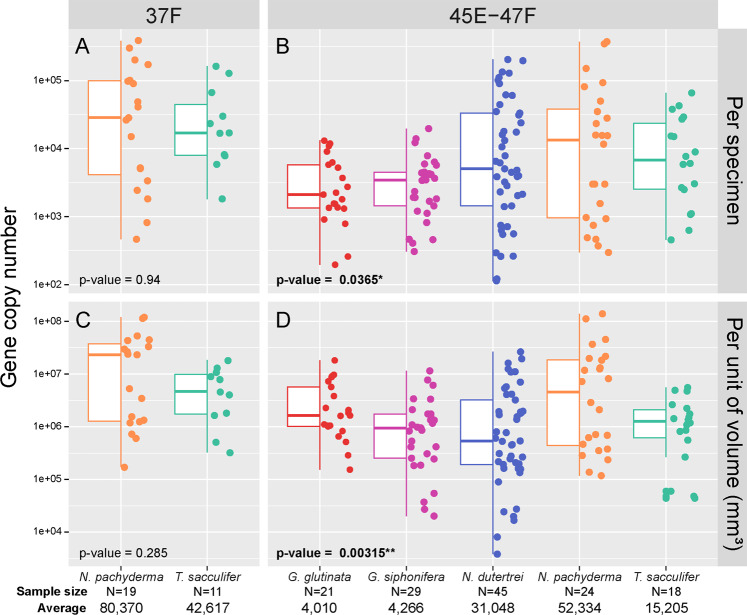
Gene copy number for the gene markers 37F and 45E-47F. Box plot and jitter plot of gene copy number per specimens (**A**, **B**) and per unit of volume (**C**, **D**) made for both markers. The number of observations per species and average gene copy number per specimen are provided below the graphs. The *p* values indicate the ANOVA results and the significant results are shown in bold with the number of asterisk giving the level of significance.

**Fig. 3:**
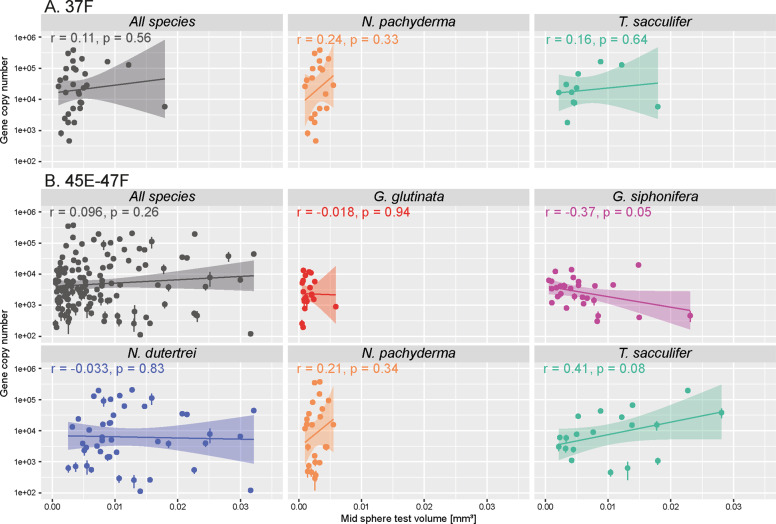
Linear regression between gene copy number and volume of individual cell for all measurements and per species. The correlation coefficient r and associated *p* values are provided for each graph.

After data curation, the first analysis consisted in evaluating the congruence between quantifications made on the 37 F and 45E–47F on specimens of *N. pachyderma* and *T. sacculifer* with a linear regression (Fig. 1). Next, we evaluated if a significant difference exists in gene copy number between species of foraminifera for individual cells (Fig. 2A, B), or between number of copies per unit of volume (gene copy numbers per mm³, Fig. 2C, D). An initial Shapiro-Wilk normality test performed on the raw data concluded that they did not follow a normal distribution (Table S1). The data were logarithmically transformed, which led to normalization of the distribution of the values for both quantification (gene copy number per specimens and per unit of volume) and markers considering all data points or individual species values except for *T. sacculifer* when considering unit volume (p-value = 0.019). We considered this as a minor deviation from normal distribution and proceeded with a one-way ANOVA (Table S2) to test for difference in means and applied a post-hoc pairwise t-test (Table S3) when the results of the ANOVA were significant. Finally, we used two complementary approaches to test for the relationship between size and number of gene copy. We ran a Two-way ANOVA to test for the interaction between size and number of gene copy (Table S4) in parallel with linear regressions between sizes of individual specimens and the number of gene copies for both markers and individual species (Fig. 3).

### Comparison with previous results and model

**Fig. 4:**
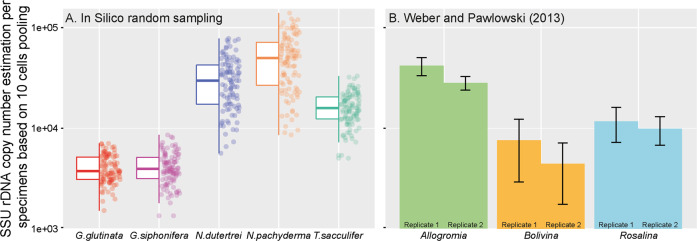
Gene copy number estimation with pooling of ten specimens. Comparison between the results of (**A**) this study and the results (**B**) of Weber and Pawlowski (2013) obtained from duplicated quantification of gene copy number of three species of benthic foraminifera based on DNA extraction of pooled specimens (*N* = 10). We performed 100 random in silico resampling of ten values obtained on single specimen.

We used a modelling approach to assess the compatibility of our results with those obtained on three benthic foraminifera species by [32]. In that study, the average number of gene copies extracted from a pool of ten specimens was quantified twice for each benthic foraminifera species. To obtain comparable results, we simulated for each species 100 random samplings of a pool of ten specimens to infer the theoretical number of gene copy that we would have obtained with a pooled approach. The gene copy number of an individual in the simulation was selected randomly (with replacement) from the observed distributions of single-cell quantifications for each species (Fig. 2).The result of this simulation is shown on Fig. 4 together with the results of [32] for direct comparison.

Next, we assessed the potential impact of the difference in average copy number between species and the large variability in number of gene copy between specimens on metabarcoding datasets. For simplicity, we simulated a theoretical population where the five species for which we have data would occur in equal proportions, and considered a population size of 50, 100, 250, 500 and 1000 specimens. We conducted 100 random samplings for each population size and calculated the theoretical relative proportions derived from a metabarcoding dataset, and with and without the application of a normalisation factor. We calculated the normalisation factor by dividing the average number of gene copy of all species by the lowest average number of gene copy (Fig. 4). All data analyses and models were performed in R [38] using ggplot2 [39] and tidyverse [40]. All data presented in the paper were made available at PANGAEA.

## Results

We successfully quantified gene copy number of 139 specimens (18 to 45 specimens per species), and for 28 specimens we obtained the gene copy number for both markers 37 F and 45E–47F (Fig. 1). Comparison of the results between the two markers revealed a high correlation (r > 0.9) for both *T. sacculifer* and *N. pachyderma*, but with an offset from the expected 1:1 line (Fig. 1). Quantifications made with the longer fragment 45E–47F systematically yielded lower number of copies but conserved the scalability across the full range of the observed gene copy number variation. Since the 45E–47F marker provides better taxonomic coverage and resolution, allowing metabarcoding of all the analysed species with the same primer pair, we chose to do the remaining quantifications with the 45E–47F marker. Using this marker the average number of gene copies per species varied from ~4000 copies in *G. glutinata* to ~50,000 copies in *N. pachyderma*. Within species, the variability was larger, ranging between 370,000 copies observed in a single specimen of *N. pachyderma* down to less than 1000 copies observed in seven specimens of this species (Fig. 2C). A similarly large variability was observed for *N. dutertrei* and *T. sacculifer*, and for *G. siphonifera* and *G. glutinata* that had an average gene copy number of ~4000, where the single-specimens values varied between ~200 and ~11,000 copies and ~300 and ~19,000 copies respectively. Hence, we observed a difference of one order of magnitude in average copy number among species, but a difference of more than two orders of magnitude among specimens belonging to the same species.

The ANOVA applied to the number of gene copies per specimen revealed significant differences when considering all species (Fig. 2, Table S2) but the pairwise comparisons showed that there only some species pairs show significant differences. Specifically, there is no significant difference between *G. glutinata* and *G. siphonifera* on the one hand, and between *N. dutertrei*, *N. pachyderma* and *T. sacculifer* on the other hand (Table S3). The same analyses applied on the number of gene copies per unit of volume (considering the actual cell volumes of the analysed specimens) returned a different picture with *N. pachyderma* being significantly different from all species except of *G. glutinata*, and *G. glutinata* being significantly different from *G. siphonifera* and *N. dutertrei* (Table S3). A significant relationship between gene copy number and cell volume appeared neither among all specimens, nor within specimens of the same species (Fig. 3), and the Two-way ANOVA also returned a non-significant *p* value for the interaction between these two factors (Table S4). Most of the relationships were positive, none of the linear regression explained more than 16% of the variance and none of the correlation coefficients were significant (Fig. 3). The results obtained with the 37 F marker were consistent with those obtained with the 45E–47F.

To connect the obtained results with the previous study by [32], we artificially pooled results from ten individuals that were quantified separately, using 100 random iterations. This approach reduced the variability such that not only the mean values of gene copy number per specimen, but also the differences among the replicates match the range of gene copy numbers observed by [32] in the benthic species *Allogromia*, *Bolivina* and *Rosalina* (Fig. 4).

**Fig. 5:**
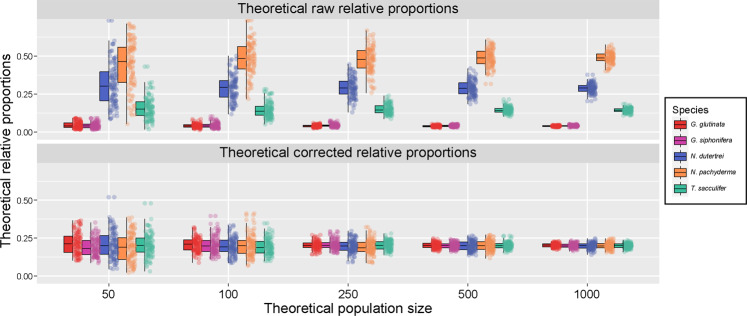
Modeling of the potential effect of gene copy number variability on metabarcoding datasets. We modelled a theoretical population where all species would occur in equal proportions, simulated five population sizes and performed 100 random samplings of the measured gene copy number and calculated the relative proportions. We applied a normalization factor on the resulting proportions to correct the difference in gene copy number to assess the impact of the variability only.

Next, using the observed magnitude of variability and observed mean gene copy number per species, we simulated metabarcoding results for a community containing the five studied species in equal proportion of individuals (Fig. 5). Modelling the effect of gene copy number variability and the average revealed that the relative proportions of each species in a metabarcoding community would strongly deviate from their “true” proportions, even where large populations were sampled (Fig. 5). Interestingly, under this configuration, species with the lowest average gene copy number (*G. glutinata* and *G. siphonifera*) would be consistently recorded as rare, even with the smallest sampled populations. By applying a correction factor based on average gene copy number per species, the metabarcoding community composition can be brought closer to the real proportions, provided enough (in this case at least 500–1000) specimens have been sampled. Applying the correcting factor equalizes the variability among the species, and the variability overall decreases systematically with increased population size.

## Discussion

Our results imply a remarkable extent of variability in gene copy number, spanning more than two orders of magnitude, within five species of planktonic foraminifera. We also show that only a small part of this variability could be explained by differences in cell volume (Fig. 3) and we did not find any evidence for a significant interaction between size and gene copy number either (Table S4). To our knowledge, this is the first demonstration of such an extent of variability among specimens of the same species. Previous studies were based on small number of replicates and although they documented large difference among species, they attributed the observed differences within species to technical bias [15, 18]. Considering the congruence between the two independent markers (Fig. 1), the observed range in gene copy numbers in our study cannot be explained by technical issues. We especially note that the direction of the observed offset between the two markers is consistent with observations that longer fragments yield lower number of copies in qPCR analyses [41]. Nonetheless, before interpreting the large variability as a primary biological signal, we consider potential other sources of bias. Having excluded technical bias of the qPCR, the only other plausible source of bias is the possibility that some of the low values observed could have been due to partial degradation of DNA in some of the analysed specimens. Ideally, for the qPCR method, the DNA should be extracted from freshly collected specimens. However, planktonic foraminifera are difficult to maintain in culture and can only be collected from the pelagic environment, which is why we analysed cryopreserved individuals from our collection [34]. Because we worked with cryopreserved specimens that had to be repeatedly manipulated for separation and then for imaging and qPCR, it is possible that the DNA could have been partly degraded, resulting in apparent lower number of gene. However, we note the mean gene copy number per species in our analyses are comparable with those of [32], who worked with living cultures (Fig. 4). If our analyses were affected by DNA degradation, the average gene copy numbers should have been lower. Hence, it is unlikely that preservation issues may have significantly affected the overall results.

Since the per species gene copy numbers are comparable to previous studies [32] and fall within the range of variability of eukaryotes, when scaled to cell volume [5], we must conclude that the observed variability reflects genuine biological differences among the sampled specimens, and that the largest part of this variability cannot be explained by differences in cell volume (Fig. 3). Our estimation of planktonic foraminifera cell is biased by two factors, the extrapolation of the measured tests semi-volume one the one hand and episodic growth in the other hand. Since all investigated species of foraminifera have a roughly comparable test morphology (trochospiral or planispiral with lobate chambers) the applied method of full test volume approximation will have affected the volume data for all species in a very similar way and did not affect the relationship between volume and gene copy number. Planktonic foraminifera grow their shells episodically by adding successive chambers [42]. Since their cell volume increases continuously, there will always be an offset between test and cell volume at any time. However, the size of this offset is unlikely larger than the volume of the terminal chamber, which would correspond among the studied species to differences smaller than a factor of two (volume of the last chamber compared to the remaining part of the shell [43]). The differences between the volume estimation and true cell volume are therefore unlikely to explain the two orders of magnitude variability in gene copy number.

Searching for other potential mechanisms explaining the variability, we note that when normalised to cell volume, the two species *G. glutinata* and *N. pachyderma* contain more gene copies than the other three species. Gong et al. (2013) suggested that there could be a positive relationship between SSU rDNA gene copy number in ciliates and the amount of intra individual variability in the gene. Both *G. glutinata* and *N. pachyderma* have extensive intragenomic variability in the SSU rDNA gene [26, 44], while *G. siphonifera* and *T. sacculifer* do not [24]. This observation points at gene conversion or a similar process occurring during replication that could lead to disproportionately large variability and number of gene copies within individual genomes. Along the similar line, we note that protists genomes are dynamic and undergo important modifications during their individual life cycles of [45]. Foraminifera are multinucleated and heterokaryotic, indicating differential genome multiplication during life, which is episodic [46], thus likely contributing to gene copy number variability per specimen in a way similar as the cell volume size bias due to episodic chamber addition as described above. However, it has also been shown experimentally that food source may influence the nuclear size and DNA content of foraminifera [47], akin to a process by which phytoplankton is able to modify its elemental stoichiometry [48]. Furthermore, prior to reproduction, the nucleus of foraminifera enlarges and DNA and other material condense into granules that are degraded in a process referred as *Zerfall* [49], allowing the foraminifera to reduce their genome back to the reproductive size [46]. During this process, the number of gene copies is likely modified and “resets” the gene copy number to a state that is independent of cell size. Finally, foraminifera can reproduce sexually or asexually [50, 51]. It results in offspring of different ploidy and likely genome organisation, potentially producing another dimension of variability in gene copy number per individual. We observe that the distribution of gene copy number seems to follow a bimodal distribution in *N. pachyderma* (Fig. 2) where asexual reproduction has been observed [50], and it could reflect expression of the diploid and haploid phases.

Although the process (or processes) controlling the variation in gene copy number in foraminifera remain elusive, the large inter individual variability we observe highlights the fact that the mentioned and further, probably unknown, cellular processes are the primary source of bias in metabarcoding studies. Our simulations indicate that the effect is greater for smaller populations (Fig. 5). Planktonic foraminifera populations are denser in the upper 100 m of the water column compared with the deeper aphotic zone, with >100 specimens per m³ in tropical and subtropical waters [52–54]. A typical depth-stratified collection of planktonic foraminifera using a multiple closing plankton net collects between five m³ of water in the surface layer and 50 m³ in the deeper layer where the population is sparse and thus recovers from a few to several hundred specimens. It is only in upwelling regions or in the highly productive temperate to subpolar areas where more than a thousand specimens would be collected at once [55]. This means that a large portion of environmental samples, especially those derived from oligotrophic regions or taken from the subsurface layer of the ocean will be affected by gene copy number variability, even when corrected for average values per species. Conversely, where the sampled populations are large, containing hundreds or even thousands of specimens (Fig. 5), the empirically derived correction factors would likely mitigate the bias due to gene copy number variation. Using a different approach [20], also showed that an in silico correction of gene copy number would affect estimates of compositionality in marine phytoplankton. Collectively, these results imply that the composition of metabarcoding, but also metagenomic, datasets can be brought closer to ecological proportionality, but due to the observed variability in gene copy number among individuals, this requires sampling of large populations.

## Conclusion

The large inter individual variability and difference in average SSU rDNA gene copy number between species of planktonic foraminifera challenges the application of metabarcoding approaches to assess community composition. Although metabarcoding offers great advantages such as covering the entire diversity in a sample including its cryptic component, the compositionality of the dataset may not reflect the actual living community in neither relative abundance or biomass. Deriving correction factors based on single-cell (or single individual) quantification or coupling metabarcoding with high-throughput imaging [56] appear to be potential avenues to characterize accurately living communities. Irrespective of this applied issue, our results highlight the dynamic nature of the foraminifera genome and hints to yet unknown cellular processes resulting in the previously unknown and unexpectedly high degree of variability in gene copy numbers among specimens of same species.

## Supplementary information

Figure S1

Table S1

Table S2

Table S3

Table S4

Supplementary Material 1
