# Supplementary figures and images for: High variability in SSU rDNA gene copy number among planktonic foraminifera revealed by single-cell qPCR
Source: ISME Commun. 2021 Oct 30;1:63. doi: 10.1038/s43705-021-00067-3 (PMC9723665; doi:10.1038/s43705-021-00067-3)

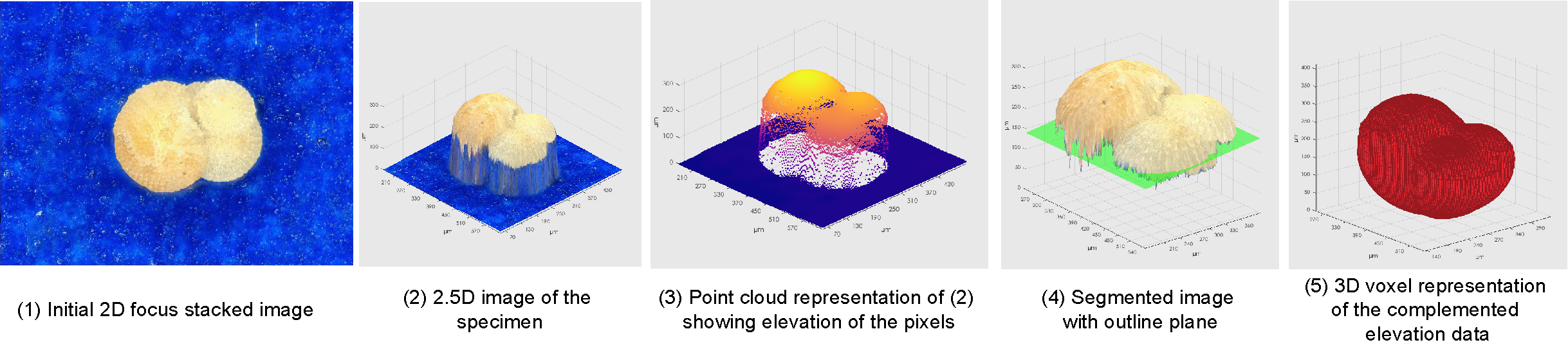

Supplement: Supplementary file 1 — Figure S1 [file 43705_2021_67_MOESM1_ESM.tif]
